# Post-fire dynamics of ectomycorrhizal fungal communities in a Scots pine (*Pinus sylvestris* L.) forest of Poland

**DOI:** 10.7717/peerj.12076

**Published:** 2021-09-15

**Authors:** Jacek Olchowik, Dorota Hilszczańska, Marcin Studnicki, Tadeusz Malewski, Khalil Kariman, Zbigniew Borowski

**Affiliations:** 1 Department of Plant Protection, Institute of Horticultural Sciences, Warsaw University of Life Sciences, Warsaw, Poland; 2 Department of Forest Ecology, Forest Research Institute, Sękocin Stary, Poland; 3 Department of Biometry, Institute of Agriculture, Warsaw University of Life Sciences, Warsaw, Poland; 4 Department of Molecular and Biometric Techniques, Museum and Institute of Zoology, Polish Academy of Science, Warsaw, Poland; 5 UWA School of Agriculture Earth and Environment, The University of Western Australia, Perth, Australia

**Keywords:** Post-fire, Mycorrhiza, Ectomycorrhizal fungal communities, Exploration types

## Abstract

**Background:**

Global warming and drying have markedly enhanced in most forests the risk of fires across the world, which can affect the taxonomic and functional composition of key tree-associated organisms such as ectomycorrhizal (ECM) fungi. The present study was conducted to characterise the alterations in the extent of root ECM colonisation, the ECM fungal communities, and their exploration types (*i.e.*, indicator of ECM soil foraging strategies) in regenerated pines within a burned site as compared with an unburned site (five years after the fire event) in the Forest District Myszyniec, Poland.

**Methods:**

To assess the ECM fungal communities of burned and control sites, soil soil-root monoliths were collected from the study sites in September 2019. A total of 96 soil subsamples were collected for soil analysis and mycorrhizal assessment (6 trees × 2 sites × 4 study plots × 2 microsites (north and south) = 96 subsamples).

**Results:**

The percentage of root ECM colonisation was significantly lower in the burned site in comparison with the unburned (control) site. However, the ECM species richness did not differ between the control and burned sites. The identified ECM species in both sites were *Imleria badia, Thelephora terrestris, Russula paludosa*, *R. badia*, *R. turci*, *R. vesca*, *Lactarius plumbeus, Phialocephala fortinii*, and *Hyaloscypha variabilis*. The most frequent species in the burned and control sites were *I. badia* and *T*.* terrestris,* respectively. The relative abundances of contact, medium-distance smooth and long-distance exploration types in the burned site were significantly different from the control site, dominated by the medium-distance exploration type in both sites. The abundance of the long-distance exploration type in the burned site was markedly greater (27%) than that of the control site (14%), suggesting that the fire event had favoured this ECM foraging strategy. The results demonstrated that the fire led to reduced ECM colonisation of Scots pine trees in the burned site whereas the species richness was not affected, which can be attributed to degrees of fire-resistance in the ECM species, survival of ECM propagules in deeper soil layers, and/or continuous entry of spores/propagules of the ECM fungi from the adjacent forests via wind, water run-off or animals.

## Introduction

Wildfires are among the main disturbance in terrestrial ecosystems, affecting aboveground and belowground biotic communities including ectomycorrhizal (ECM) fungi (Bond & Keeley, 2005; Bond, Woodward & Midgley, 2005). Ecosystem services provided by ECM fungi are diverse and include improved succession and re-establishment of forest trees, solubilisation of mineral nutrients (Courty et al., 2010), alleviation of biotic/abiotic stresses such as soil-borne pathogens and drought (Shah et al., 2016), and modification (oxidative decomposition) of soil organic matter to facilitate the decomposition process by soil saprotrophs, and improvement of CO_2_ fertilization effect (Terrer et al., 2016; Kariman, Barker & Tibbett, 2018). The heat generated by fire generally only affects the surficial soil profile, but the extreme temperatures caused by fire in the upper soil layers can cause changes in soil physicochemical and microbial properties (De Bano, 2000). Moreover, the continuity of the ECM-host interactions can be disrupted due to loss of vegetation (host plants), which is crucial for survival of ECM fungi as they generally possess very limited saprotrophic capacity and are highly dependent on living host trees as the carbon source (Shah et al., 2016; Zak et al., 2019).

Many ECM fungal species show some degree of host specificity at the level of host plant family or genus (Smith & Read, 2008). Hence, post-fire changes in the vegetation type (*i.e.,* loss of certain ECM host plants) may substantially affect the ECM fungal community composition. The composition and structure of the ECM fungal communities after return to the pre-fire conditions require many years (Torres & Honrubia, 1997; Stendell, Horton & Bruns, 1999; Grogan, Bruns & Chapin, 2000; Dahlberg, 2001; Treseder, 2004; Bastias et al., 2006; Rincón & Pueyo, 2010; Kipfer et al., 2011). Some studies have shown that fire can have negative, neutral or positive effects on fungal diversity (Dove & Hart, 2017). Furthermore, the extent of post-fire root ECM colonisation was shown to be decreased (Dhillion, Anderson & Liberta, 1988; Rashid et al., 1997; Barker et al., 2013), increased (Herr et al., 1994; Rincón et al., 2014), or unaffected (Eom et al., 1999). These variable responses of ECM fungi to fire are often attributed to differences in fire severity and frequency among study sites (Cairney & Bastias, 2007; Hart et al., 2005).

Since the fire’s impact and heat decrease rapidly from the topsoil downward, the detrimental temperatures only occur in the uppermost layer (De Bano, 2000). Kipfer et al. (2010) conducted artificial soil heating experiment and found that some ECM species (*Cenococcum geophilum*, *Rhizopogon roseolus* and *Wilcoxina rehmi*) survived the heat, suggesting there might be considerable resistance to fire among the ECM fungal species. Following disturbance, the ECM fungal species may have high re-colonisation abilities because of their abundant stock of resistant propagules (*e.g.*, spores, sclerotia) in the soil (Taylor & Bruns, 1999; Rincón & Pueyo, 2010), and possibly due to survival of some ECM hyphal networks and colonised roots located in deeper soil layers that are not usually affected by the fire. Study of key ECM-related factors such as root colonisation, species richness, and exploration types are required to improve our knowledge on the survival and functionality of ECM fungi in burned forest sites.

The main aim of the present study was to characterise the differences in the structure and recovery of ECM fungal communities associated with pines in burned and unburned sites (Forest District Myszyniec, Poland) five years after the fire event.

## Materials and Methods

### Study sites

The selected forest sites dominated by Scots pine (*Pinus sylvestris* L.) were located in north-east Poland in Forest District Myszyniec. The climate of this area is classified as temperate, with large amplitudes of annual temperatures, sudden changes in seasons, and low rainfall. The average annual temperature is 7.4 °C, the average amount of precipitation: 485–527 mm, the growing season lasts 185–200 days. Hypsometrically, the area is not very diverse. The dominant feature is the flat outwash plain, which forms the southern part of Masurian Sander. It consists of sand, which in some places forms dunes up to 20 m high (Borowski et al., 2020).

In May 2014, a high-intensity wildfire burned 96 ha of a *P. sylvestris* forest, while 1,500 ha of the forest remained undisturbed. The average stand age of the forests in which the fire occurred was 40 years. The estimated temperatures of flame combustion reached 1,000–1,200 °C and flameless eruption about 400–500 °C. The mean soil temperature during the forest fire at several centimetres’ depth varied between 100 and 150 °C. However, due to the large heterogeneity of fire severity, tree mortality in the burned stands varied between 50% and 60%. Within the study area, burned and control sites were at least 500 m apart. To avoid the edge effect, both sites were located at least 100 m from the nearest forest edge and 200 m from the edge of the burned area. Details of the selected study sites and their soil characteristics are given in Table 1. Both the experimental (burned) and the control plots (not affected by fire) were established in a forest dominated by pine (90%) with a small admixture of *Betula pendula* (10%). Other tree or shrub species—such as *Padus serotina*, *Quercus robur*, *Juniperus communis*—were also occasionally present at the sites studied. The most common woody species in the lower layers of the stand on unburned plots were common juniper, Scots pine, and Pedunculate oak. Average species cover in this zone was very low, not exceeding 1%. In burned plots, the most common species was Silver birch (19% cover), then American bird cherry (4% cover) and Scots pine (1% cover).

**Table 1:** Geographical location and soil chemical properties of the study sites (Forest District Myszyniec, Poland). Letters indicate significant differences between sites at p <0.05 (Tukey’s test; *n* = 25).

	**Control**		**Burned**	
Localization	53,212013° N; 21,366840° E		53,212581° N; 21,383340° E	
Forest type	Dry coniferous forest		Dry coniferous forest	
Soil type	Endoskeletic Albic Podzols (Arenic)		Endoskeletic Albic Podzols (Arenic)	
pHH_2_O	4.3	a^a^	4.4	a
N-NH_4_ (mg/l)	12.0	a	12.2	a
P (mg/l)	6.9	a	5.9	a
K (mg/l)	23.9	a	22.5	a
Ca (mg/l)	97.4	a	80.4	b
Mg (mg/l)	12.5	a	11.0	b
Cl (mg/l)	35.9	a	32.1	b
C_org_ (%)	0.7	a	0.7	a

### Experimental design, sample collection and processing

To assess the ECM fungal communities of burned and control sites, soil-root monoliths of about 15  × 15  × 15 cm were collected from the study sites in September 2019. At each site, four replicated 10  × 10 m plots separated more than approximately 200 m were selected in a haphazard fashion for sampling roots. Two soil subsamples were taken from each of 48 trees (6 trees from each study plot), one sample from the northern side and one from the southern side of the tree bases (*i.e.,* each sample consisted of two microsite localities with the same type of vegetation: north and south). A total of 96 soil subsamples were collected for soil analysis and mycorrhizal assessment (6 trees × 2 sites × 4 study plots ×2 microsites = 96 subsamples). At the time of data collection on the study plots located in the experimental area (burned forest), all burned trees were still present (no logging) and some of them were felled. Additionally, due to the lack of other mature tree within the 100 m^2^ study plots, we could be sure that we were only sampling Scots pine roots. In the laboratory, soil samples were carefully sieved to separate roots from the soil. Roots were subsequently washed with tap water.

### Morphological characterisation and molecular identification of ECM fungi

The clean root fragments were examined with a Zeiss Stemi 2000-C stereomicroscope (Carl Zeiss, Germany; ×10–60 magnification) for ECM colonisation. The root tips were classified as “non-vital ECM” (NV) “vital non-ECM” (NM) and “vital ECM” (VM) as previously described in Olchowik et al. (2020). Viable ectomycorrhizal root tips were further classified into morphotypes based on their morphological characteristics (Agerer, 1991). Three to four representative root tips from each morphotype were stored at −20 °C to await further identification by molecular methods. The identification of selected morphotypes was based on molecular analysis of two to three ECM root tips of each unique morphotype. The ITS-1 and ITS-4 primers sets, the universal primers for ECM community studies, were used to amplify the ITS-1, 5.8S and ITS-2 regions of the fungal nuclear ribosomal DNA (White et al., 1990; Gardes & Bruns, 1993). The amplified products were sequenced using the Sanger sequencing method. Molecular identification of the ECM species was performed as described previously (Olchowik et al., 2020). The reference sequence with the highest sequence similarity percentage (98%–100% in most cases; Table 2) with the query sequence was used for identification. Additionally, the ECM species were classified into the exploration types given by (Agerer & Rambold, 2004–2015)

**Table 2:** Estimated species richness, diversity, and occurrence of ectomycorrhizal fungal taxa associated with the roots of Scots pine trees in control and burned sites. Data are the frequency (Freq.; percent of trees colonised by a given ectomycorrhizal (ECM) species) and abundance (Abun.; percent of ECM roots colonised by a given ECM species) of fungal taxa associated with roots of Scots pine. NCBI (National Center for Biotechnology Information), Identity (the extent to which two sequences have the same residues at the same positions in an alignment). Different superscript letters indicate significant differences between treatments (Tukey’s test, *p* < 0.05).

	**BLAST top-hit**			**Control**		**Burned**	
**Identification**	Exploration type	NCBI	Identity [%]	Freq.	Abun.	Freq.	Abun.
**Basidiomycota**							
*Thelephora terrestris*	medium distance smooth	MT469917	100	88	19.9	63	11.3
*Imleria badia*	long distance	MT469918	99	63	8.3	71	10.1
*Russula paludosa*	contact	MT469919	98	38	3.0	38	2.9
*Russula badia*	contact	MT469920	100	29	4.0	21	1.4
*Lactarius plumbeus*	contact	MT469921	99	25	2.4	33	3.7
*Russula turci*	contact	MT469922	100	17	1.2	17	0.8
*Russula vesca*	medium distance smooth	MT469923	98	13	1.2	4	0.2
**Ascomycota**							
*Phialocephala fortinii*	–	MT469924	99	25	7.2	33	4.6
*Hyaloscypha variabilis*	–	MT469925	98	21	5.1	38	1.6
Degree of mycorrhization [%]				52.3^a^		37.1^b^	
**Estimated species richness**					
Chao1				3.2^a^		3.2^a^	
**Diversity**					
Shannon–Wiener (H’)				0.8^a^		0.7^b^	

### Soil chemical analysis

Twelve soil cores were collected from each study site. The soil analyses were performed in the laboratory of the Polish Centre for Accreditation No. (AB312) following their established protocols. For each soil sample, pH was measured by mixing 20 ml of soil substrate with 100 ml of deionized water and 1 M KCl, respectively (ISO10390, 1997). The exchangeable cations (Ca, Mg, K) were quantified following the ISO 11260 (2011) protocol. The chloride was determined following the ISO 7393-2. The soil phosphorus (P) concentration was determined following extraction with 1% citric acid (Schlichting, Blume & Stahr, 1995).

### Data analysis

The significant of differences in the abundance of ectomycorrhizal taxa with different exploration types between both study sites were calculated use one-way generalised linear model (GLM). For this GLM model we assumed binomial distribution with log link function for response variables which were relative abundance of ectomycorrhizal taxa with each study exploration types. For soil chemical properties we used *t*-test for evaluation the difference between control and burned sites. Nonmetric multidimensional scaling ordination (NMDS) based on the Bray–Curtis distance matrix was used to illustrate the differences between the abundance of ECM fungal species in both study sites. To test the significative of these differences, we used analysis of similarities methods (ANOSIM). The ANOSIM is non-parametric statistical method, used to tests whether we can reject the null hypothesis that the similarity of the abundance of ECM fungal species between control and burned site is greater than or equal to the similarity within the control or burned site. We used redundancy analysis RDA to determine which soil chemical properties were the most significant to explain variation of the abundance of ectomycorrhizal taxa with different exploration. Results of RDA was visualized on biplot figure. The Chao-1 and Shannon–Wiener’s (H ′) parameters were calculated based on the abundance and numbers of the ECM fungal species. To test difference between control and burned sites for these two parameters we used *t*-test, previously the assumption of normality of the distribution was checked. The all considerate statistical methods we used data for individual trees, because there was no auto-correlation between the trees within the study plot. However, for soil chemical properties where was collected two subsamples for individual trees, the results for two subsamples were averaged. Statistical analyses were performed using R version 3.6.1 with the vegan package. The accepted level of significance was *p* < 0.05.

## Results

### Soil properties

Soil properties of the two study sites are summarized in Table 1. Both control and burned sites had acidic soils (pHH_2_O = 4.3 and 4.4, respectively). The concentrations of available macronutrients (NPK) and total organic carbon did not differ significantly between soils of control and burned sites, whereas soil from the control site had significantly higher concentration of Ca, Mg, and Cl compared to those of the burned site.

### ECM fungal communities of the study sites

**Figure 1:**
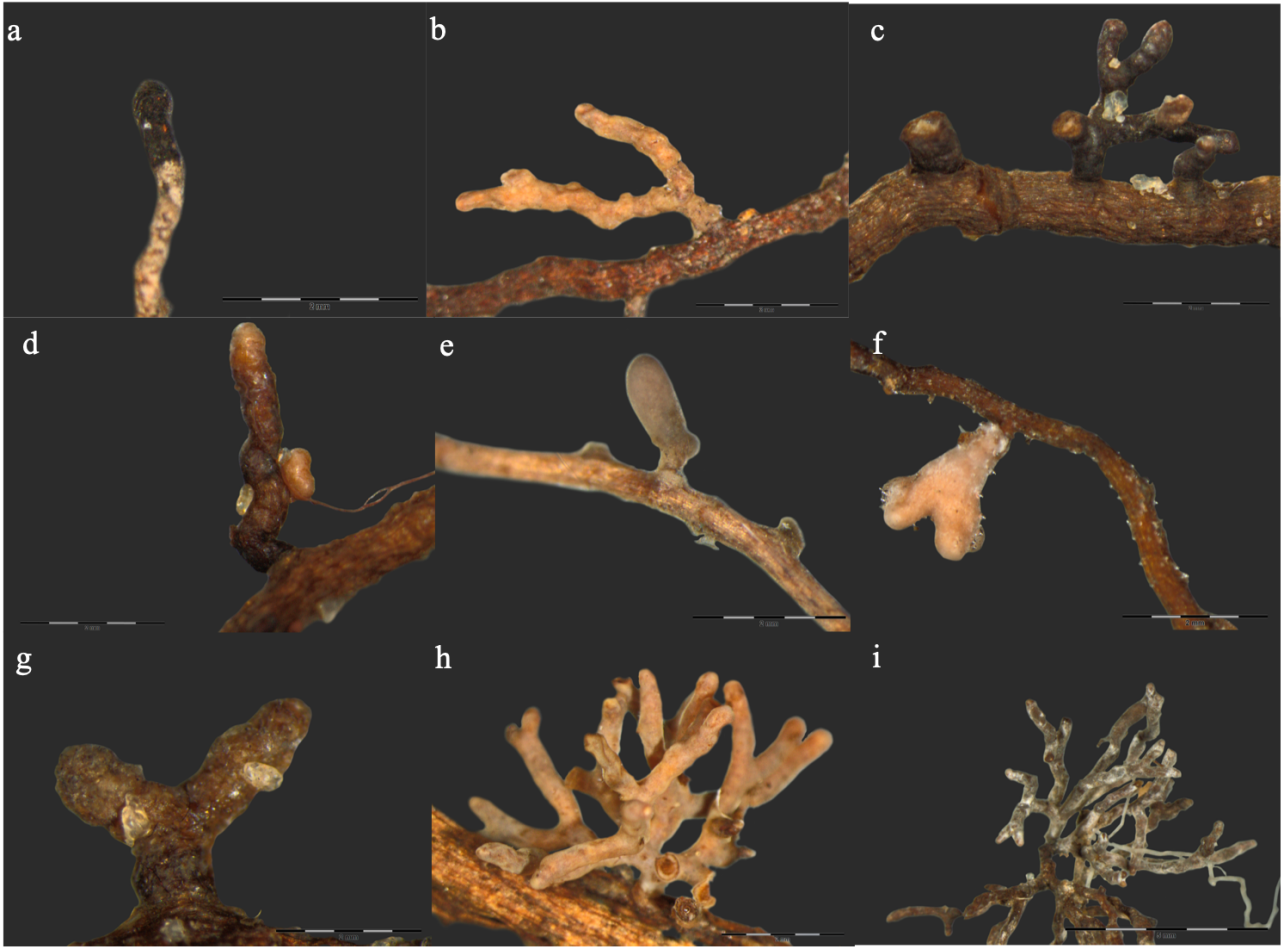
Ectomycorrhizal root tip morphotypes formed by different fungal species in roots of Scots pine trees. (A) *Hyaloscypha variabilis* (Hambl. & Sigler) Vohník, Fehrer & Réblová, (B) *Lactarius plumbeus* (Bull.) Gray, (C) *Phialocephala fortinii* C.J.K. Wang & H.E. Wilcox, (D) *Russula badia* Beeli, (E) *Russula paludosa* Britzelm., (F) *Russula turci* Bres., (G) *Russula vesca* Fr., (H) *Thelephora terrestris* Ehrh., (I) *Imleria badia* (Fr.) E.-J. Gilbert. Bars in each photograph indicate 0.4 mm length.

Morphological features of the ECM root tips formed by the identified ECM species is presented in Fig. 1. Direct sequencing of the DNA obtained from distinguished morphotypes revealed a total of 9 ECM fungal taxa in the roots of examined Scots pines (Fig. 1, Table 2). The ECM species richness did not differ between control and burned site (Table 2). However, the percentage of ECM root colonisation was significantly lower in the burned site as compared to the control site. The most frequent ECM species in the burned site were *Imleria badia* (Freq. = 71%) and *Thelephora terrestris* (Freq. = 63%). In the control site, however, *T. terrestris* was the most frequently occurring ECM species (88%), followed by *I. badia* (63%). Beside *I. badia* and *T. terrestris*, the ECM fungal communities of both forest sites were composed of *Russula paludosa*, *R. badia*, *R. turci*, *R. vesca*, *Lactarius plumbeus, Phialocephala fortinii* and *Hyaloscypha variabilis.* The Shannon–Wiener diversity estimator predicted respective values of 0.8 and 0.7 species for control and burned sites, respectively (Table 2).

**Figure 2:**
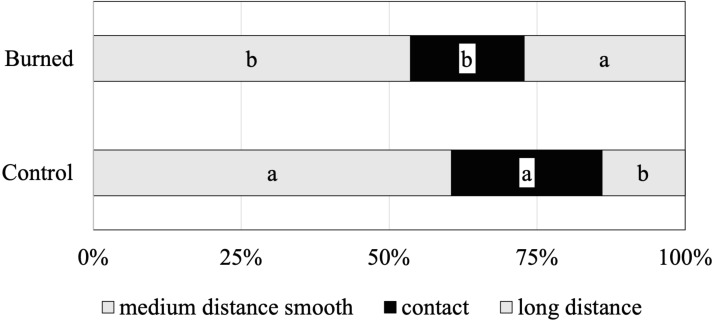
Mean relative abundance of ectomycorrhizal taxa with different exploration types associated with Scots pine trees in control and burned sites. Within each root tip classification, different letters indicate significant differences between sites (Tukey’s s contrast, *p* = 0.05).

The functional diversity of the identified ECM species was assessed by subclassifying morphotypes into different exploration types (indicating the ECM foraging strategies) (Fig. 2). The relative abundances of contact, medium-distance smooth and long-distance exploration types were significantly different between control and burned sites. The ECM fungal community in both burned and control sites was clearly dominated by fungal species of the medium-distance exploration type (61% and 54%, respectively). The abundance of the long-distance exploration type was greater in the burned site (27%) than that of the control site (14%) (Fig. 2).

**Figure 3:**
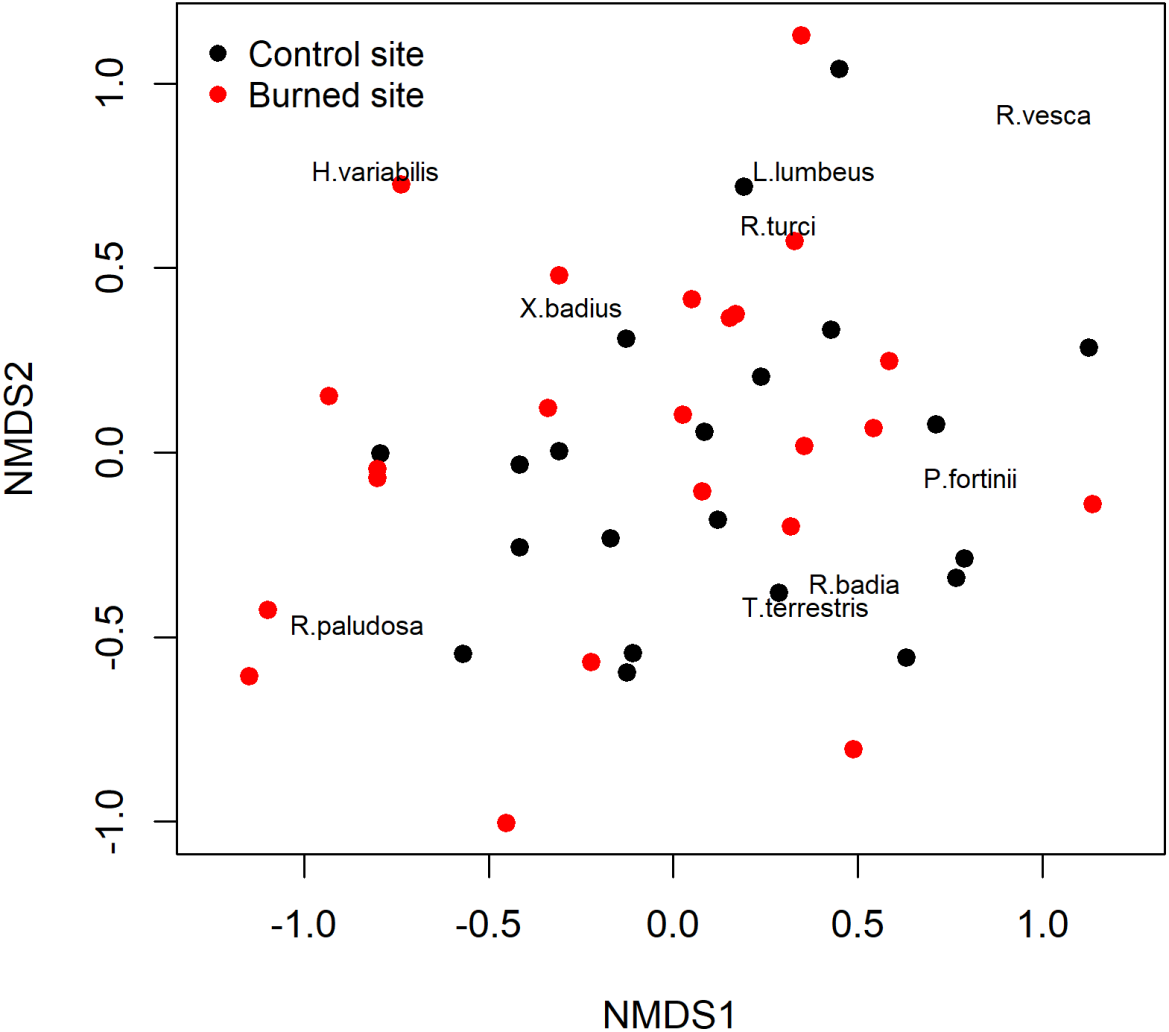
Non-metric multidimensional scaling (NMDS) of the study sites based on their ectomycorrhizal fungal communities. Black: burned site; red: control site.

The non-metric multidimensional scaling (NMDS) ordinations analysis did not separate the ECM fungal assemblages for and burned sites. In other words, samples from the burned site did not differ in their ECM species composition from those of the unburned site as demonstrated by the NMDS (Fig. 3), which was further confirmed by the analysis of similarity ANOSIM (*p* = 0.3926).

**Figure 4:**
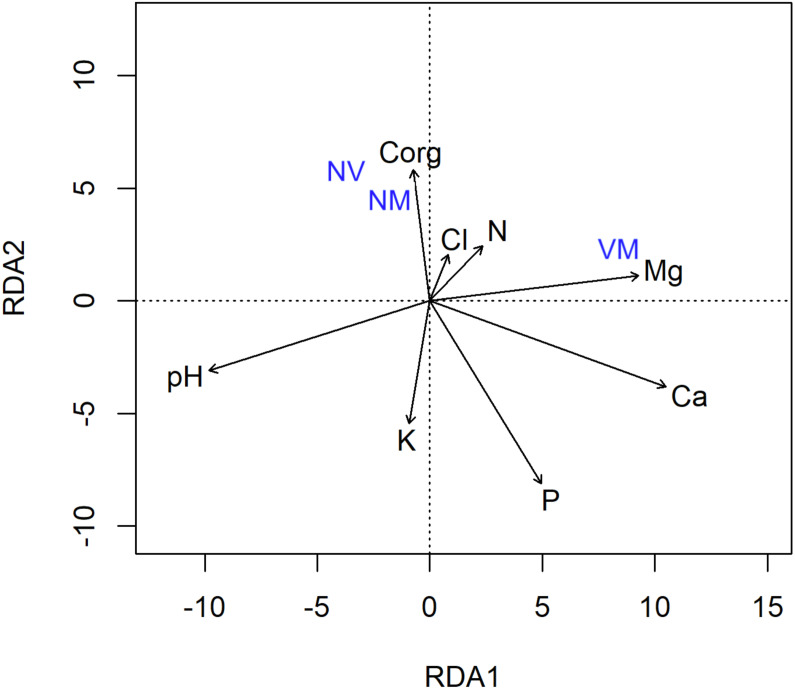
Redundancy analysis (RDA) of the abundance of different ectomycorrhizal root tip types vital mycorrhizal (VM), non-vital (NV) and vital non-mycorrhizal (NM) associated with Scots pine trees in relation to soil chemical properties.

The redundancy analysis (RDA) with forward selection selected soil characteristics as the explanatory variables, significantly explaining the variation in the abundance of different ECM root tip types (Fig. 4), where the VM root tip category was positioned separately, and away from the NV/NM categories. The final model explained 10.7% of the variation. The RDA also revealed a strong correlation between the abundance of ECM root tips and soil acidity (*F* = 2.71) (Fig. 4). The soil acidity arrow on the RDA diagram was positioned away from the VM root tips, indicating that presence of VM root tips was negatively correlated with soil pH. Furthermore, VM root tips were clustered close to Mg and N-NH_4_, possibly reflecting an effective functionality of the ECM fungi.

## Discussion

Given the ongoing global climate change, the enhanced frequency and intensity of fire events across the globe is inevitable for the 21st century (Pechony & Shindell, 2010; Flannigan et al., 2013; Moritz et al., 2014). The presence of ECM fungi is vital for the successful post-fire establishment of Scots pine trees (Molina, Massicotte & Trappe, 1992). Hence, the majority of research dealing with the impact of fire on ECM symbiosis is focused on *Pinus*-dominated ecosystems (Taudiére, Richard & Carcaillet, 2017).

In our study, trees from the burned site had reduced ECM colonisation, which can be due to the negative effects of fire (Table 2). Similarly, in a study conducted by Longo, Urcelay & Nouhra (2011) the ECM colonisation of *Nothofagus pumilio* trees was found to be lower in the burned site (five years after the fire event) compared to the unburned site. However, according to the meta-analysis performed by Dove & Hart (2017), fire did not significantly affect the extent of mycorrhizal colonisation of plants across a diverse array of studies. To promote the establishment of young tree seedlings in disturbed sites, we need to ensure the seedlings have high level of ECM colonisation prior to planting, which can be achieved in advanced plant nurseries through diverse inoculation techniques including mycelia or spore-based inoculations (Repáč, 2011). Disturbed sites might have unfavourable soil conditions that need to be modified by organic amendments such as biochar in order to achieve higher mycorrhizal colonisation in young seedlings (Warnock et al., 2007).

Results of the present study demonstrated that the ECM species richness of Scots pine trees was not negatively impacted in the burned site, which is in line with several other studies (Jonsson et al., 1999; Grogan, Bruns & Chapin, 2000; Rincón & Pueyo, 2010; Longo, Urcelay & Nouhra, 2011). However, richness of post-fire ECM fungal communities was shown to be reduced in some other studies (Dahlberg, 2001; Kipfer et al., 2011; LeDuc et al., 2013). The reduced ECM species richness likely contributes to the decreases in the post-fire ecosystem processes (Dore et al., 2010; Holden, Gutierrez & Treseder, 2013; Toberman et al., 2014). Nevertheless, the magnitude of the ECM species richness response may also depend on the functional redundancy of the soil microbial community (Nielsen et al., 2011). According to Dove & Hart (2017) ECM species richness after fire have rather short-term effect and recovery to pre-fire condition occurs in one or two decades. Inconsistent results have been achieved about the impact of fire on ECM richness, depending on the host tree species, the severity of fire, the elapsed time after fire, and the local post-fire edaphic-climatic conditions (Nielsen et al., 2011). Species richness is a poorly informative proxy of the fire impact on ECM fungal communities because the same richness indices may hide very different fungal community compositions (Wilsey et al., 2005). Lack of reduction in the number of ECM species in our study (Table 2) may indicate that either the identified ECM species survived the fire and re-colonised newly formed roots after the fire, (ii) some ECM propagules (such as resistant spores, extramatrical mycelia, sclerotia, and rhizomorphs) survived in deeper soil layers that are less affected by fire (Johnson & Gehring, 2007; Claridge, Trappe & Hansen, 2009), and/or iii) new ECM fungal spores/propagules entered the sites *via* wind (Koske, 1987; Trappe & Luoma, 1992), water run-off (Trappe & Luoma, 1992) or small mammals (Jonsson et al., 1999; Pyare & Longland, 2001). Resilient ECM communities with the ability to rapidly re-colonise tree roots as well as the entry of ECM spores/propagules from the adjacent areas are crucial for successful post-fire pine recruitment (Kipfer et al., 2011; Taylor & Bruns, 1999).

Numerous ECM fungal species are sensitive (in terms of abundance) to abiotic shifts and climatic conditions that affect both their vegetative and reproductive structures (Kranabetter, Durall & MacKenzie, 2009). For instance, LeDuc et al. (2013) identified many late-stage fire-sensitive ECM species in the *Cortinarius* and *Russula* genera. In our study, we observed decreased abundance of four *Russula* species including *R. paludosa*, *R. badia*, *R. turci* and *R. vesca* in the burned site. The abundance of *T. terrestris* within the post-fire site significantly declined, which is in contrast to that observed for the *Pinus banksiana* trees in the Canadian boreal forest (LeDuc et al., 2013). A variety of contrasting responses has been documented among co-occurring ECM species following fire events (Taudiére et al., 2017).

Our analysis of the ECM exploration types revealed that the identified ECM species varied in terms of their putative functional/ecological roles. In the burned site, the ECM fungal communities were dominated by the medium-distance smooth types (Fig. 2), represented by species such as *T. terrestris* and *R. vesca*. However, the species belonging to long-distance exploration types have been previously reported to be the dominate ECM species associated with pine roots in post-fire areas (Horton, Cázares & Bruns, 1998; Baar et al., 1999; Rincón & Pueyo, 2010; Buscardo, 2010). Nevertheless, the long-distance exploration type was greater (nearly 2-fold) than that of the control site in our study, which is partially in agreement with the previous studies indicating that fire stimulated the occurrence of the long-distance exploration type (Baar et al., 1999; Rincón & Pueyo, 2010; Buscardo, 2010). The long-distance exploration types, due to their dispersal abilities, are more adapted to colonise less compact root systems (Peay, Kennedy & Bruns, 2011). As the roots obtained from the burned site had fewer vital root tips (Table 2) than those from the unburned site, it also had a higher proportion of long-distance ECM types (Fig. 2). The ECM species *I. badia* was clearly favoured in the burned site, suggesting that its long-distance exploration type mycelia may have conferred competitive advantages to this species.

The changes in soil chemistry (*e.g.*, pH and nutrient availability) may influence the belowground allocation of plant photosynthates to root fungal symbionts (Dove & Hart, 2017). As tree mortality deprived ECM fungi of different sources of C substrates, soil-related aspects were associated with contrasting responses of the fungal community. In our study, the soil from the burned site showed significant reductions of Ca, Mg and Cl (Table 1), which might be among the factors contributing to the reduced extent of root colonisation or alterations in abundance/frequency of the identified ECM species. In study by Hilszczańska, Gil & Olszowska (2019) lower values of carbon, nitrogen and some others nutrients have been observed in burned plots a year after fire. Similar changes involving the washing out of organic compounds deeper into the soil profile are often observed (De Bano, 2000; Certini, 2005). The RDA analysis also supports this hypothesis, as a strong negative correlation between soil pH and elements in soil (*i.e.,* Mg, Ca and Cl) was unravelled (Fig. 4). Furthermore, the VM root tips were clustered close to Mg and N-NH4, which could be due to ECM-mediated increases in soil nutrients availability and promoted N2 fixation activity of free-living diazotrophs, respectively (Paul, Chapman & Chanway, 2007).

Pérez-Izquierdo et al. (2021) in their study showed, that the survival of *P. sylvestris* after fire plays a key role in maintaining fungal communities (Pérez-Izquierdo et al., 2021). In our study, survival of *P. sylvestris* after fire (50% to 60% among the burned stands) could played a key role in maintaining ECM fungal community. Most of the ECM fungal species that survived in burned stand in our study belong to common in fungal spore banks in boreal ecosystems (Glassman et al., 2015). Because the ectomycorrhizal fungi can persist on root tips of surviving trees after a fire, they could serve as effective strategy for post-fire establishment, growth and survival of trees (Smith & Read, 2008).

## Conclusions

The results demonstrated that the Scots pine trees in the burned site had reduced level of ECM colonisation. The ECM species richness, however, was similar in control and burned sites (five years after the fire), presumably due to degrees of fire-resistance in the identified ECM taxa, survival of ECM propagules in deeper soil layers and/or entry of viable ECM spores/propagules from adjacent forests *via* wind, water run-off or animals. Further studies are required to determine the long-term impact of fire on the composition and functionality of ECM fungi in fire-affected forests, and improve our understanding of the main edaphic factors driving the fire-related alterations in ECM composition, frequency, and functional traits (*e.g.*, exploration types).

##  Supplemental Information

10.7717/peerj.12076/supp-1 Supplemental Information 1 All mycorrhizal species and soil parameters These data were used for statistical analysis to compare study sites.

## Additional Information and Declarations

### DNA Deposition

The following information was supplied regarding the deposition of DNA sequences: The best representatives of each unique ITS sequence are available at the NCBI GenBank: MT469917–MT469925.

### Data Availability

The following information was supplied regarding data availability: The raw data are available as a Supplementary File.
